# Resolving laminar activation in human V1 using ultra-high spatial resolution fMRI at 7T

**DOI:** 10.1038/s41598-018-35333-3

**Published:** 2018-11-20

**Authors:** Sriranga Kashyap, Dimo Ivanov, Martin Havlicek, Shubharthi Sengupta, Benedikt A. Poser, Kâmil Uludağ

**Affiliations:** 1 Department of Cognitive Neuroscience, Faculty of Psychology and Neuroscience, Maastricht University, 6229 EV Maastricht, Netherlands; 2 Center for Neuroscience Imaging Research, Institute for Basic Science (IBS), Department of Biomedical Engineering, Sungkyunkwan University, Suwon, 16419 Republic of Korea

## Abstract

The mesoscopic organization of the human neocortex is of great interest for cognitive neuroscience. However, fMRI in humans typically maps the functional units of cognitive processing on a macroscopic level. With the advent of ultra-high field MRI (≥7T), it has become possible to acquire fMRI data with sub-millimetre resolution, enabling probing the laminar and columnar circuitry in humans. Currently, laminar BOLD responses are not directly observed but inferred via data analysis, due to coarse spatial resolution of fMRI (e.g. 0.7–0.8 mm isotropic) relative to the extent of histological laminae. In this study, we introduce a novel approach for mapping the cortical BOLD response at the spatial scale of cortical layers and columns at 7T (an unprecedented 0.1 mm, either in the laminar or columnar direction). We demonstrate experimentally and using simulations, the superiority of the novel approach compared to standard approaches for human laminar fMRI in terms of effective spatial resolution in either laminar or columnar direction. In addition, we provide evidence that the laminar BOLD signal profile is not homogeneous even over short patches of cortex. In summary, the proposed novel approach affords the ability to directly study the mesoscopic organization of the human cortex, thus, bridging the gap between human cognitive neuroscience and invasive animal studies.

## Introduction

The human cortex is structured into a myriad of structural and functional units^1^. These units can be indexed along both the normal and tangential coordinates with respect to the surface of the cortex^2^. The normal coordinates represent the cortical depth, from the pial to the white-matter boundary (WMB), also called cortical layers or laminae. The tangential coordinates represent the distance along the cortical ribbon, and the structural-functional units along this axis are called brain areas and columns, on the macroscopic and mesoscopic scales, respectively. These subdivisions are characterized by their distinctive histological profiles^3,4^ or by their specific responsiveness to external stimuli and role in cognitive and physiological processes^5,6^. Specifically, functional units are typically assessed in animal models using invasive methods, such as electrophysiological recordings^7^ and intrinsic signal optical imaging^8^. Based on invasive animal studies, a microcircuit model of the cortex has been proposed with differential roles of cortical layers in feed-forward processing of stimuli and cognitive feed-back^9^. In contrast, human cognitive neuroscience usually assesses only macroscopic functional organization due to limitations in spatial resolution. For example, data using functional magnetic resonance imaging (fMRI), the most popular non-invasive human neuroimaging technology, are typically acquired at 1.5 and 3T with voxel dimensions of around 3 mm, which is about the average cortical thickness.

FMRI has proven to be an invaluable non-invasive imaging tool for mapping brain areas involved in cognitive and/or sensory processing^10^ or resting-state activity^11^. It typically utilizes the blood oxygenation level-dependent (BOLD) contrast^12,13^ to probe neuronal activity indirectly via changes in blood oxygenation and cerebral blood volume (CBV)^14^. The increased availability of ultra-high field (UHF) human MRI scanners (≥7T) and the development of novel fMRI acquisition techniques (see Poser and Setsompop^15^ and references therein) have led to routinely achieving sub-millimetre spatial resolutions and thus, allow the non-invasive examination of laminar structures responsible for functional processing in humans (for reviews, see Lawrence, *et al*.^16^, Dumoulin, *et al*.^17^). Most UHF fMRI studies in humans probing laminar circuitry thus far have utilised the BOLD signal as the functional contrast^18–27^. Recently, in addition to the BOLD contrast, CBV measurements using the VASO technique have also been utilised to study functional laminar profiles in the human cortex^28^. Other contrast mechanisms, such as using cerebral blood flow (CBF) and oxygen metabolism (CMRO_2_), for laminar fMRI in humans are also currently under development (for a recent review see Huber, *et al*.^29^). Currently, all these alternatives to GE-BOLD have lower signal-to-noise (SNR), contrast-to-noise (CNR), spatial resolution and brain coverage.

However, data acquisition and analysis challenges for high-resolution fMRI remain, which hamper the direct interpretation of the measured fMRI signal in terms of layer- and columnar-specific neuronal activity. Firstly, although fMRI is the non-invasive technique with the highest spatial resolution, the voxel size (e.g. 0.7–0.8 mm isotropic) still is large compared to the spatial dimensions of cortical layers and columns. Consequently, to infer the underlying layer-specific fMRI signal, the functional data are typically spatially upsampled using various interpolation techniques and averaged over an extended region-of-interest (ROI)^30^. This approach demands very high accuracy in segmentation of the cortical sheet and co-registration of anatomical-functional data and relies on the assumption that the depth-dependent fMRI signal along the cortical distance (i.e. tangential direction) is identical within the ROI. An alternative approach is the sorting of voxels according to the relative distance of their centroids from the cortical boundaries^25,30^ (see Discussion, Effective spatial resolution). Secondly, the BOLD signal stems from changes in blood oxygenation (and CBV) mainly in the post-capillary vascular compartments. Cortical ascending veins drain the blood from individual cortical layers towards the cortical surface, which creates contamination of the local layer-specific fMRI signal with non-local signal changes stemming from the lower layers (i.e. closer to the WMB). This leads to a BOLD signal spatial profile^21,22,31^ remarkably incongruent to electrophysiological laminar profiles^14^.

Please note that the current work focuses on the first acquisition-related issue, with the aim of achieving ultra-high spatial resolution capable of directly resolving the laminar fMRI signal and thus, removing the need for upsampling and averaging over an extended ROI. To address the second physiology-related issue, several modelling attempts^32–34^ have been proposed to remove the contribution of the non-local effects due to ascending veins to the measured (steady-state or dynamic) fMRI signal.

**Figure 1:**
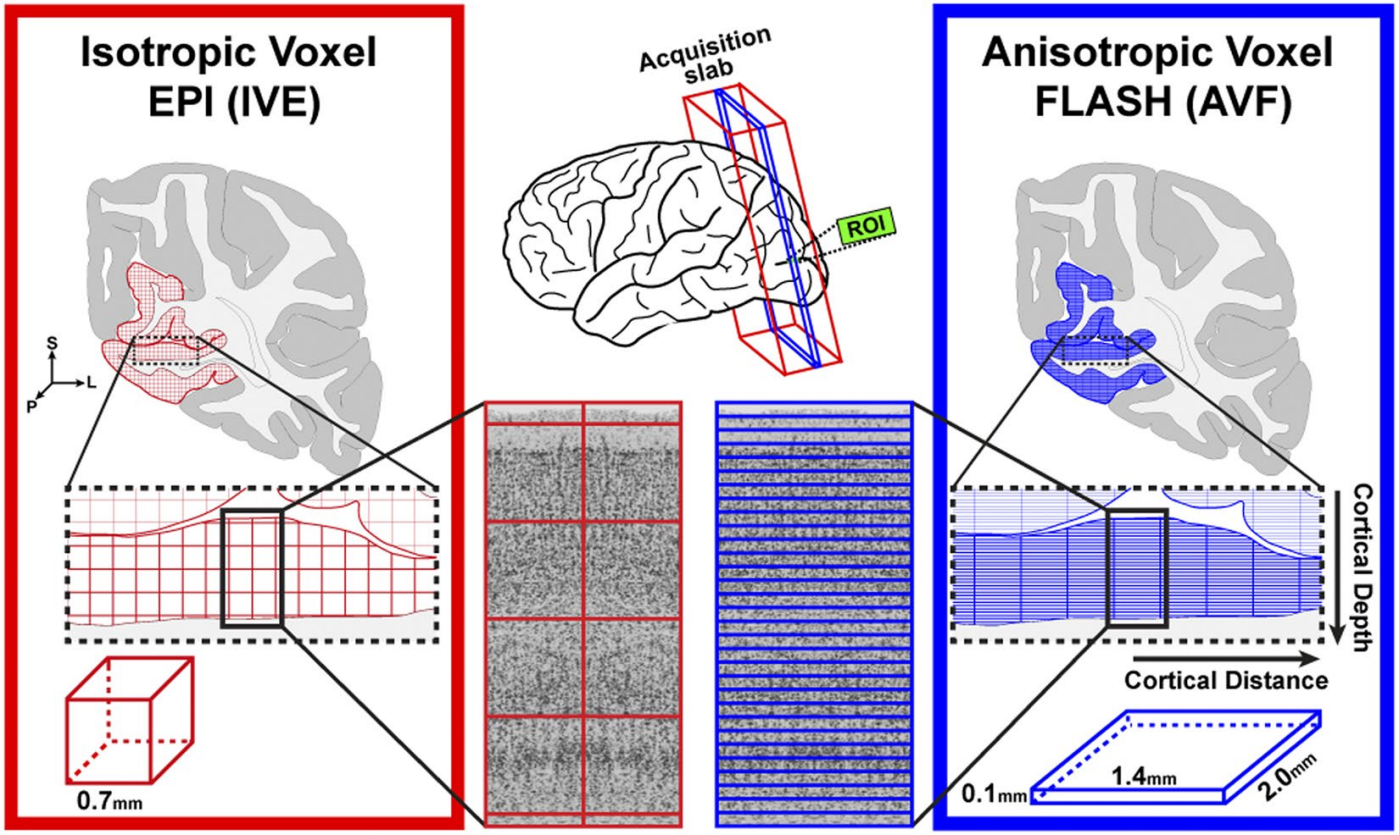
Illustration of the IVE (left, red) and the novel AVF (right, blue) acquisition schemes for layer-specific fMRI, highlighting the differences in the sampling of cortical depths between the two approaches.

Inspired by the line-scanning fMRI in animals^35^, this study showcases a novel layer-specific fMRI acquisition strategy in humans at 7T that can sample the cortical depths at an unprecedented spatial resolution (i.e. 0.1 mm) using unique highly anisotropic voxels (Fig. 1, right). We demonstrate the feasibility of the novel Anisotropic Voxel FLASH (AVF) acquisition to obtain laminar- and columnar-specific fMRI signals in the human visual cortex. Furthermore, we show that the layer-specific BOLD signal mapped by the AVF within a single ‘voxel-column’ is highly stable and can thus, reliably capture layer-specific changes in small patches along the tangential cortical distance. As layer-specific profiles can be obtained without averaging over an extended ROI, we are able to detect, for the first time, a remarkably high variability of laminar profiles along the cortical distance, which is difficult to detect with the standard Isotropic Voxel EPI (IVE) and even with invasive electrophysiology. Finally, it is generally believed that the IVE acquisitions can, in principle, achieve any effective super-resolution desired via post-processing steps if there are enough voxels within the ROI and each of the sub-millimetre voxels sample the cortical layers depths in a quasi-random fashion, i.e., there is a spatial jittering of the voxels relative to the cortical layers. We argue and demonstrate using simulations that the effective laminar spatial resolution of the IVE acquisition is much lower than the AVF, even if an extended ROI is utilized to derive cortical depth profiles.

Nevertheless, we do not propose the AVF approach as a standalone, but rather as a supplemental tool that can be used to aid the interpretation of the depth-dependent or lower-resolution signal changes observed with isotropic sub- or supra-millimetre acquisitions, typically used in cognitive neuroscience applications. Taken together, this study demonstrates the feasibility of directly assessing the laminar and columnar cortical organization in humans, non-invasively, using ultra-high spatial resolution fMRI, a scientific domain previously reserved for invasive animal studies.

## Results

The current study has two main goals: (1) To demonstrate the feasibility of cortical depth sampling using AVF. In order to show this, we compare the depth profiles obtained with AVF with those of IVE, typically utilized in laminar studies. (2) To examine the functional properties of AVF for studying cortical depth and distance profiles and using simulations, establish the superiority of the effective spatial resolution of AVF in two dimensions compared to IVE, albeit with lower brain coverage.

### Feasibility of anisotropic imaging for laminar fMRI

**Figure 2:**
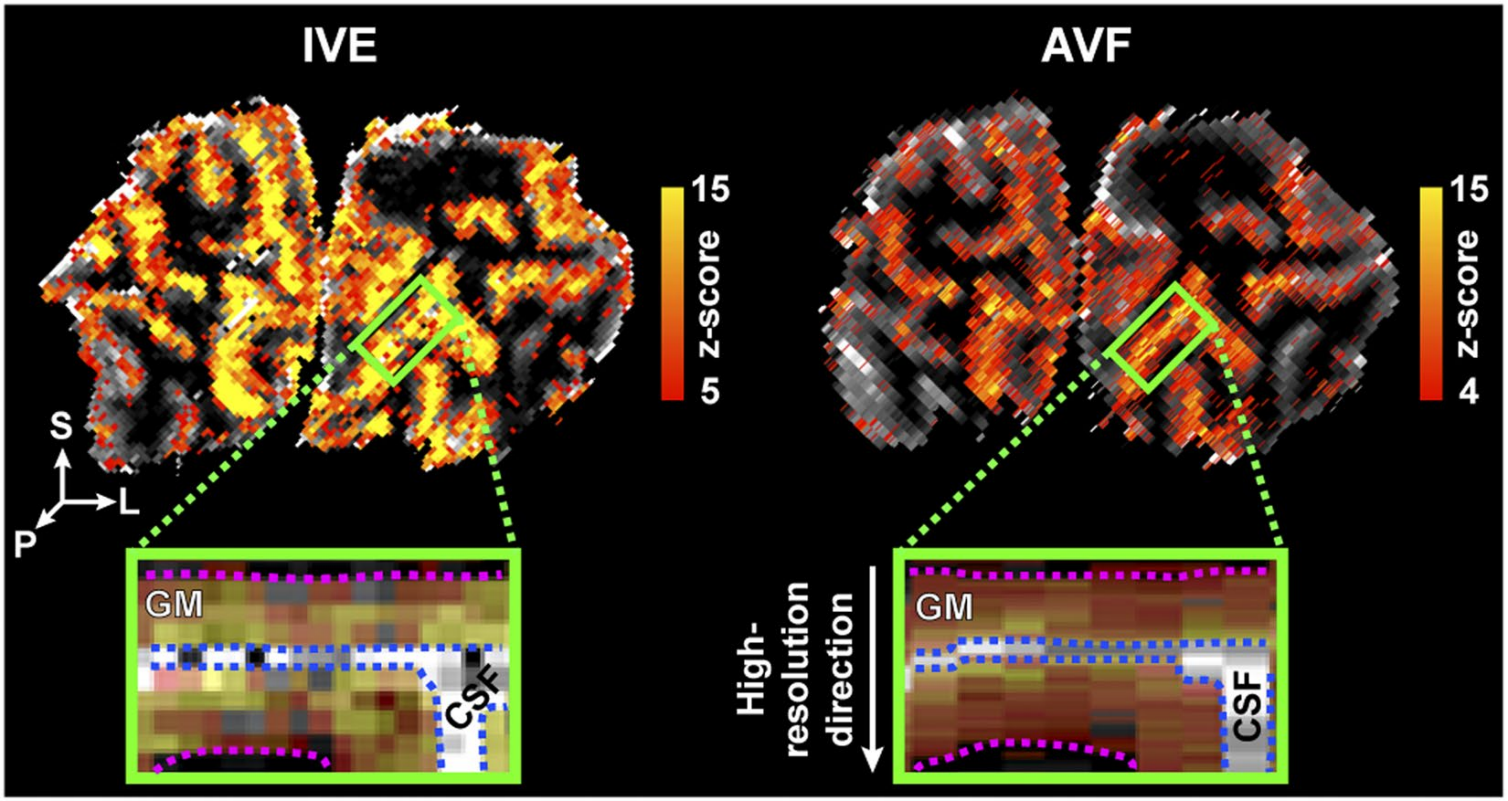
Example of single-subject, single-run activation maps for the flickering checkerboard stimulus, overlaid on the MI-EPI and the MP2RAGE T_1_ maps for the IVE (left) and AVF (right) acquisitions, respectively. The green rectangle indicates the ROI in a coronal slice through the occipital lobe. In the zoomed-in panels, the dotted pink line indicates the GM-WM border and the blue line the GM-CSF border. In the activation maps, the values for CSF are masked out to better visualize the ‘flat’ cortical patch of interest.

In both the IVE and AVF acquisitions, activations in V1 were robustly detected in all subjects within single runs (illustrated in Fig. 2 left and right, respectively). In Fig. 2, the zoomed-in panel illustrates an example ROI, showing the “flat” portion of the calcarine sulcus that was used for cortical depth analysis. The z-scores obtained from the GLM analysis are lower in the case of the AVF data, which is expected given the higher tSNR of the IVE data, partially due to the slightly larger voxel volume.

**Figure 3:**
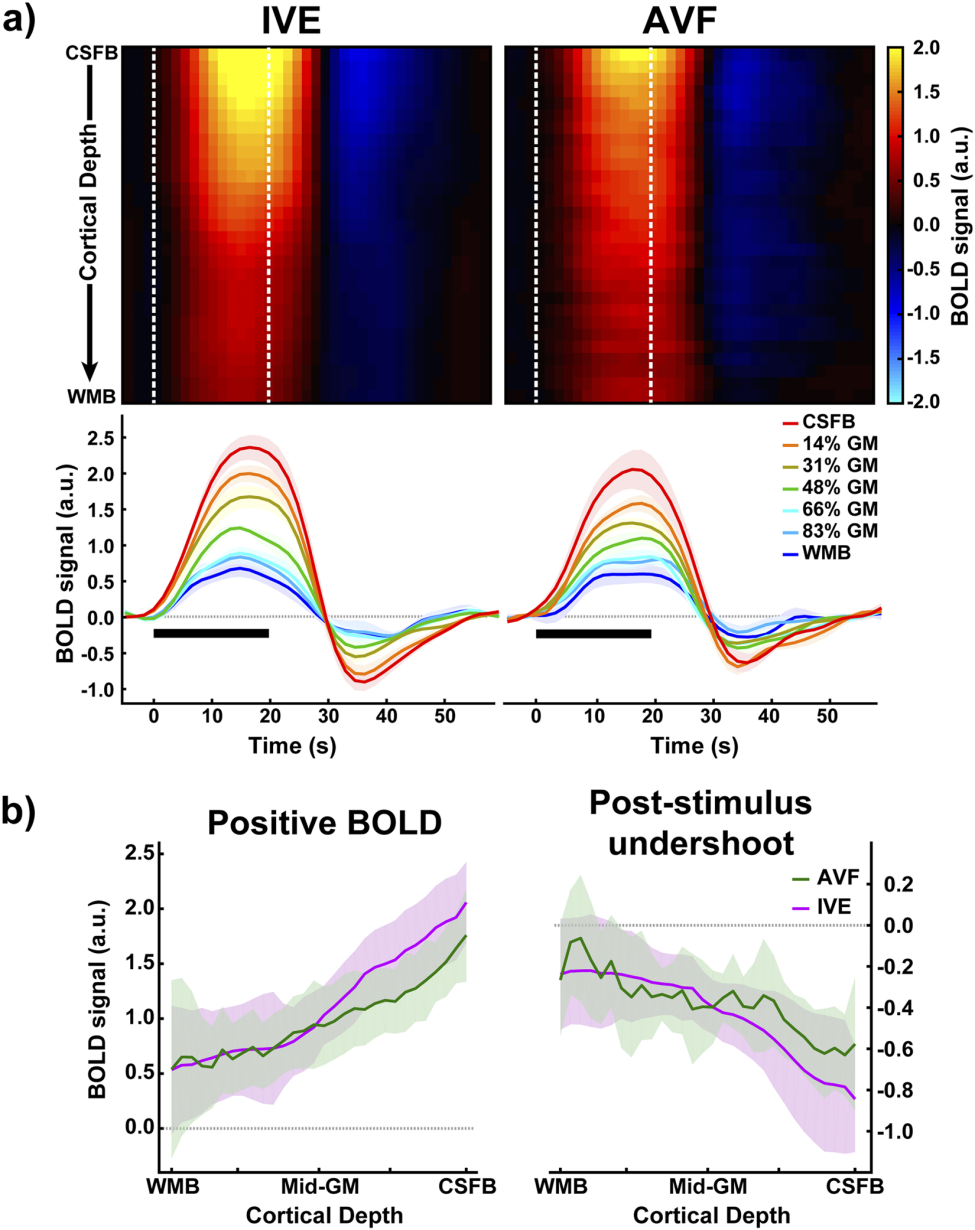
(**a**) Top: Colour-map representation of the average hemodynamic timecourses for 30 cortical depths from the IVE (left) and the AVF (right) acquisitions from CSF boundary (CSFB) to the white matter boundary (WMB). The white dotted lines on the colour-map indicate the stimulus-on period. Seven of the 30 cortical depth timecourses are presented below the heatmaps. (**b**) Cortical depth profiles of the average positive BOLD signal and post-stimulus undershoot for the two acquisition schemes. The BOLD signals have been normalized (see SI Methods) and the shaded regions represent the standard error of the mean (SEM).

The cortical depth timecourses for both acquisitions exhibit the typically observed steady BOLD signal amplitude increase towards the cerebrospinal fluid (CSF) boundary (Fig. 3) (see Uludaǧ and Blinder^14^ for a recent review). In addition, strong post-stimulus undershoots are observed^21,24^ also increasing towards the CSF boundary. A statistically significant difference in the most superficial 36.7% of grey-matter (GM) can be observed between the positive BOLD cortical depth profiles (Fig. 3b, left) obtained with the AVF compared to the IVE (F (29, 174) = 65.6, p_FDR_ < 0.0001). The post-stimulus undershoot profiles (Fig. 3b, right) also exhibit a statistically significant difference (F (29, 174) = 8.231, p_FDR_ < 0.0001) between the two acquisition approaches, albeit only in the most superficial cortical depth.

### BOLD variability along the cortical distance

**Figure 4:**
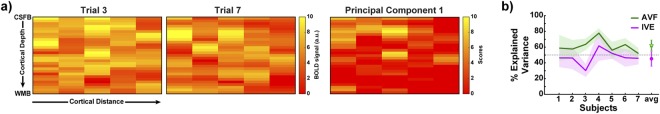
(**a**) (left to right) Average positive BOLD signal in the ROI of an example subject is shown for two trials in a single AVF run. The third panel shows the first PC obtained using PCA decomposition across all trials. (**b**) The percentage of variance explained by the first PC for all 7 subjects is plotted for the two acquisition approaches. The star and circle represent the group average percentage explained variance. The shaded regions and error bars indicate the standard error of the mean (SEM).

An interesting and consistent finding for the AVF data is the variability of the cortical depth profiles along the tangential cortical distance. Although the stimulus evokes a strong response in all voxels along the cortical distance, neighbouring voxels can be remarkably different, an observation, which is difficult to make using the IVE approach (or even using invasive electrophysiology). Thus, we investigated further whether this tangential variability stems from thermal noise (random) or is reproducible due to underlying (neuronal or vascular) physiological processes. The high degree of similarity between the patterns of positive BOLD signal profiles in an ROI from two example trials within a single AVF run is illustrated in Fig. 4a. The tangential variability exhibits a high level of agreement between the two trials and, following the Principal Component Analysis (PCA) decomposition across trials, the first Principal Component (PC) retains the tangential variability. This suggests that the tangential variability in the AVF data is reproducible, cortical variability. The percent variance explained by the first PC from the AVF data for the individual subjects (see Fig. 4b) is significantly higher compared to that of the IVE data (means: 61.3 ± 8.31% and 48.9 ± 9.32%, respectively, t (11.79) = 2.708, p = 0.0193), which was unexpected given the lower tSNR of the AVF data.

### Effective spatial resolution of the isotropic acquisition

**Figure 5:**
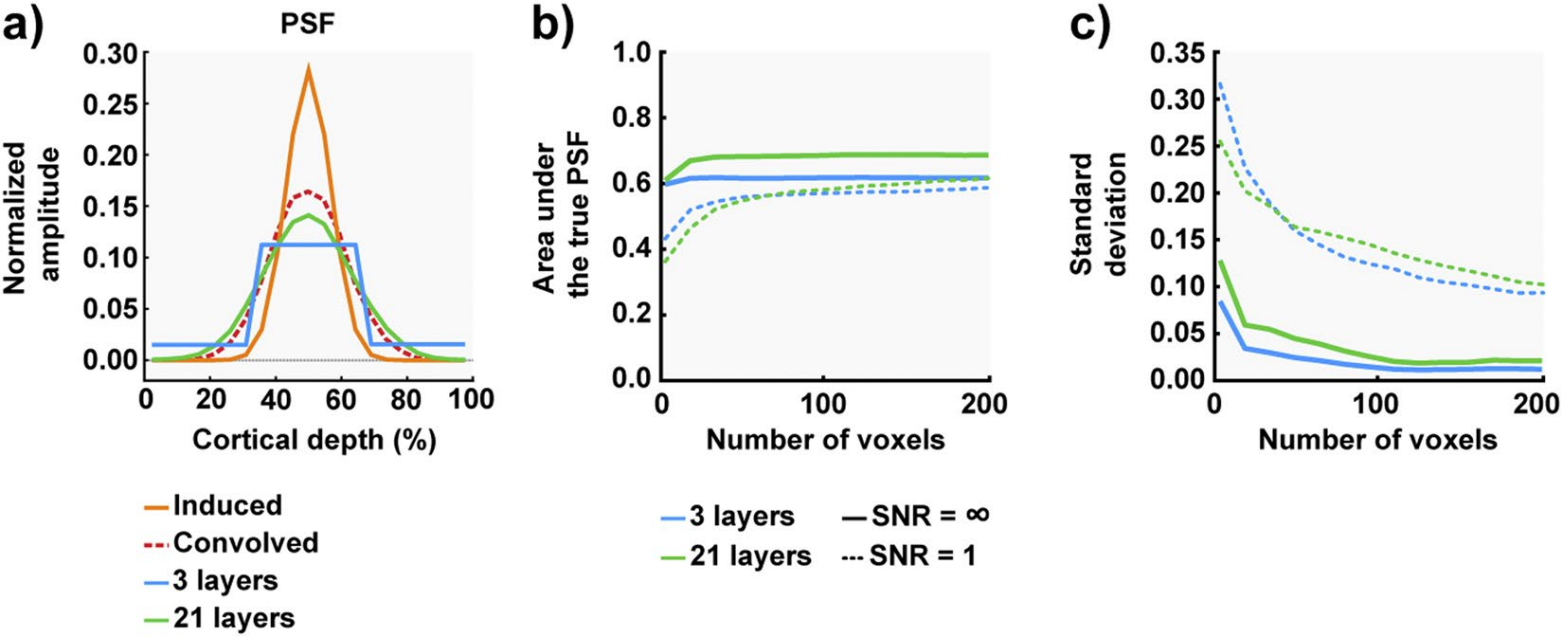
(**a**) The induced PSF used in the simulation (FWHM ~1/5^th^ of the cortical depth) is plotted together with the reconstructed PSFs obtained by convolving with kernel derived for the 0.75 mm voxels described in Koopmans *et al*.^25^, sampling 3, 21 cortical depths with infinite SNR and large number of voxels (1000). (**b**) The overlap between the induced and reconstructed PSFs with the two different sampling strategies is quantified as a function of ROI size (i.e. number of voxels, profiles of 200 out of 1000 voxels are displayed) at two different SNR levels (ideal = infinite, realistic = 1). (**c**) The standard deviation of the reconstructed PSF from the induced PSF with increasing number of averaged voxels.

Figure 5a compares the induced PSF to the average reconstructed PSF for different number of sampled layers (simulated for the case of infinite SNR and maximum number of voxels). Figure 5b shows the dependence of the mean area under the induced PSF on the number of voxels. It reveals that, for the case of infinite SNR, relatively small number of voxels (~24, e.g. 4 depth x 8 distance voxels) are sufficient to reconstruct 61–68% of the induced PSF, with a higher percentage associated with the higher number of sampled layers. However, when simulated with added noise (SNR = 1; i.e. realistic for our fMRI data), more voxels are needed to reliably reconstruct the layer-specific activation profile. With less than 80 voxels (corresponding to ~20 voxels along the tangential cortical distance), lower number of layers are preferable, albeit with a very high standard deviation (Fig. 5c), hence resulting in low reliability of the estimated PSF. With ROIs larger than 100 voxels, sampling with higher number of layers can benefit the estimation of the underlying PSF. Importantly, even for the ideal case (SNR = ∞), increasing the size of the ROI (>100 voxels) only minimally improves the estimated PSF, suggesting that the IVE acquisition, combined with standard analysis using upsampling of the functional data, is unable to fully recover the underlying physiological profiles.

### Mapping the BOLD signal along cortical distance with AVF acquisition

**Figure 6:**
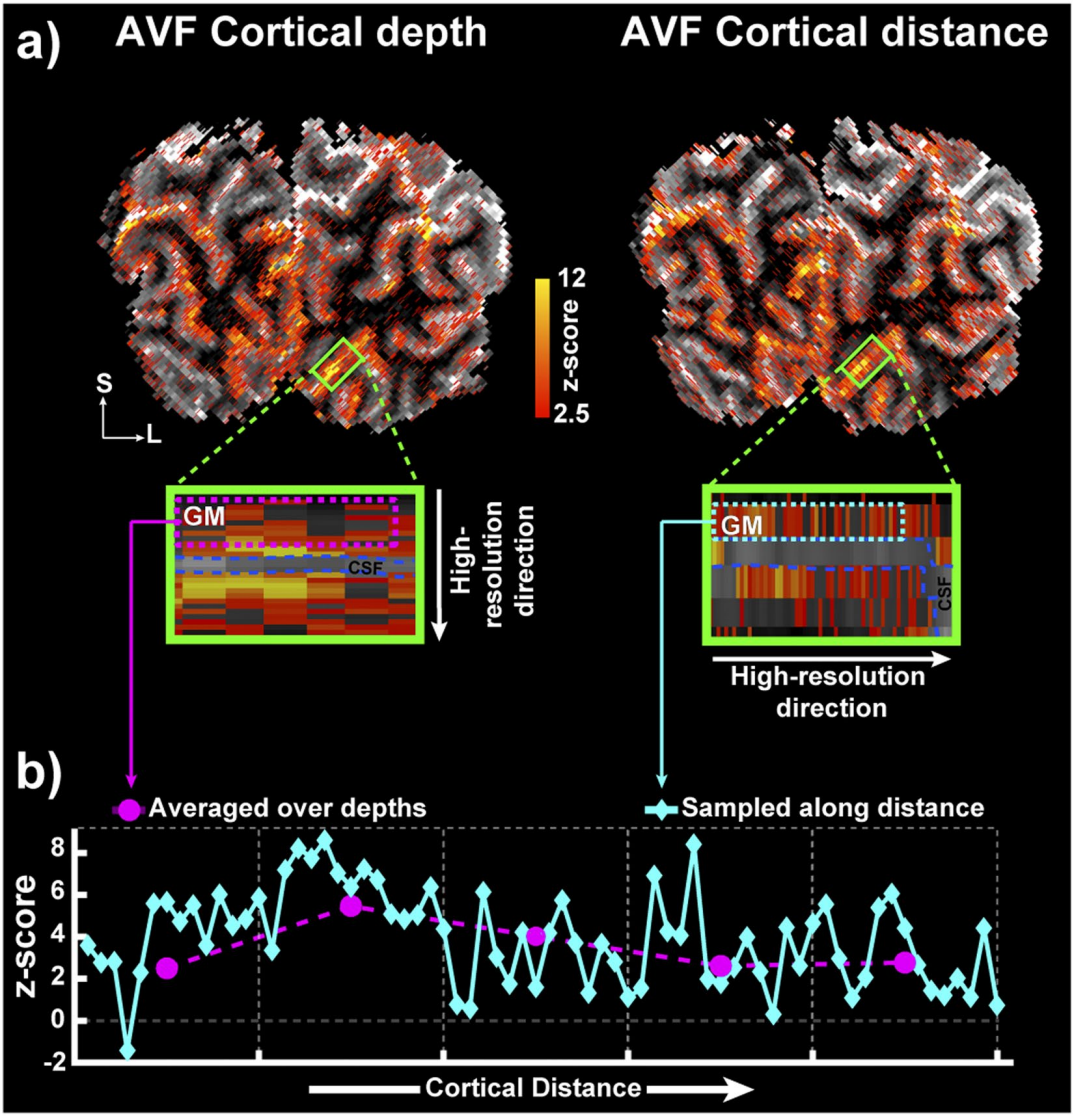
(**a**) Single-subject, single-run statistical activation map overlaid on the MP2RAGE T_1_ map with the highest spatial resolution (0.1 mm) oriented along the cortical depth (left) and cortical distance (right). The green rectangle represents the ROI, and the zoomed-in panels highlight the high-resolution sampling of cortical depth and distance, respectively. (**b**) BOLD activation z-scores are plotted from the regions in magenta and cyan dotted lines in the zoomed-in panels from (**a**).

Figure 6a shows a high degree of similarity in the single-subject, single-run statistical activation maps induced with the flickering checkerboard stimulus for acquisition with the highest resolution in the cortical depth (left) and cortical distance (right) directions, respectively. The z-scores from the regions demarcated in magenta (“laminar” acquisition) and cyan (“columnar” acquisition) dotted lines in the zoomed-in panels (Fig. 6a) are plotted in Fig. 6b. The magenta circles represent average z-score of 14 depths (a patch of 1.4 mm) in the high cortical depth resolution acquisition from the same rectangular patch of cortex as sampled with the high cortical distance resolution acquisition. The cyan diamonds represent the z-score with a higher spatial resolution along cortical distance (0.1 mm). The data obtained with “laminar” acquisition closely tracks those of the average “columnar” acquisition. This adds further credence to the finding observed in Fig. 4 that the tangential variability observed is not simply thermal noise but is most likely of physical or physiological origin. Interestingly, the BOLD activation z-scores along the cortical distance (cyan diamonds) change by two or three times, even over 0.1–0.2 mm.

## Discussion

High-resolution fMRI has provided researchers the ability to map cortical depth-dependent BOLD signal changes allowing addressing neuroscientific questions at the mesoscopic scale in humans. Cortical depth-dependent fMRI studies typically acquire voxel sizes of 0.7–0.8 mm isotropic (Fig. 1, left), with the isotropicity considered to be indispensable due to the gyrification of the human cortex. However, as the cortex is only 2–4 mm thick, even such high spatial resolution results in large partial volume effects over laminae and also with white matter (WM) and cerebrospinal fluid (CSF).

In this study, we demonstrated the feasibility of non-invasively acquiring laminar fMRI data in humans at an unprecedented spatial resolution of 0.1 mm. To that end, we utilized a T_2_^*^-weighted FLASH sequence^36^ with highly anisotropic voxels and compared the cortical depth profiles with the standard isotropic acquisition using EPI. The AVF approach presented in this study marks (at least) an 85% increase in spatial resolution in the cortical depth direction with fMRI compared to typical sub-millimetre acquisitions and remains relatively unaffected by image distortions due to the FLASH readout while having robust and reliable tSNR. An in-house developed visual cortex coil^37^ (see Supplementary Fig. S1) was used in this regard for practical advantages, such as an extended visual field for stimulus presentation and improved temporal signal-to-noise (tSNR) throughout the occipital lobe. A relatively low read-out bandwidth of 60 Hz/px was chosen to improve image SNR of the AVF data at the 26 ms TE and off-resonance effects can be a concern. It is important to note that dynamic off-resonance effects are mostly dominated by field changes due to movement. However, in the absence of deliberate motion, as was the case with the current study, their effect is a small fraction of the voxel size in the frequency encoding direction^38^ and therefore, has negligible impact on the results.

Please note that for this study we used a highly anisotropic acquisition (0.1 × 1.4 × 2.0 mm^3^), whose spatial dimensions can be adjusted if desired. That is, other voxel dimensions, e.g. voxels with 0.3 × 1.0 × 1.0 mm^3^ having approximately a similar voxel volume, can be used with the same MR sequence. Such voxels still enable to probe laminar fMRI activation without the need for spatial interpolation and at the same time, having the other dimensions only slightly larger than in standard laminar studies. Finally, as the choice of high-resolution direction is up to the investigator, we envision that multiple high-resolution directions can be used and thereby allowing the underlying high-resolution activation pattern to be inferred. In a future study, we will explore this possibility.

The current study relies on T_2_^*^-contrast using GE acquisition and therefore, suffers from the same vascular biases as isotropic GE acquisitions. GE-BOLD signal has been theoretically predicted (Uludag, *et al*.^39^, Uludaǧ and Blinder^14^ and references therein) to have high sensitivity to local vascular and metabolic changes that are tightly coupled to the regional neuronal activation^40^, but its tissue and laminar specificity is reduced due to the inherent bias of the GE-BOLD signal to both ascending and pial draining veins. This has also been consistently observed experimentally by mapping the GE-BOLD signal across cortical depths^20,21,25,27,31,41^, wherein the GE-BOLD signal increases towards the pial surface, inconsistent with laminar profiles observed using invasive electrophysiology. Several modelling efforts have tried to address the issue of venous bias in the GE-BOLD data in the context of laminar fMRI^22,32–34^ but still require validation with invasive electrophysiology.

Approaches, such as SE-BOLD and GRASE-BOLD acquisitions, also suffer from the ascending vein effect because of a) direct contribution of ascending veins to the T_2_-contrast and b) an additional T_2_* decay during read out^42^, albeit to a lesser degree compared with GE-BOLD acquisition^14,39^. Alternatively, other fMRI techniques, such as VASO (measuring CBV) or ASL (measuring CBF), are promising candidates to study cortical depth profiles without the unidirectional spatial blurring associated with the ascending veins. However, total CBV contrast may be associated with point-spread function extending beyond the locus of neuronal activation due to a) unidirectional spatial blurring in the direction of the penetrating arteries: It has been shown that, for short stimulus duration, the largest CBV change is in the arteries and they can dilate retrogradely in the upper layers with respect to location of neuronal activation; b) significant CBV changes in veins for long stimulus duration (for a recent review, see Uludaǧ and Blinder^14^). Currently, our novel anisotropic approach is limited to GE-BOLD contrast, but future work can expand it to these alternative contrasts.

In both acquisition types investigated in this study (i.e. isotropic vs anisotropic), we confirmed the increase of the BOLD signal amplitude towards the GM-CSF boundary when considering the same ROI. Differences between the acquisitions could be observed at the depths close to the GM-CSF boundary (Fig. 3b), possibly due to a) the inherent smoothing of the IVE acquisition, or b) contamination of signal changes due to non-local susceptibility effects stemming from the draining pial veins. With regard to a), the higher signal increase near the pial surface with the IVE acquisition may originate from the larger point-spread function, thereby leading to greater partial voluming with surface veins. In this regard, Monte Carlo simulations were carried out to simulate the effect of leakage of the signal in the pial vein to the cortical layers due to partial volume effects on an anatomically-inspired cortex^43^ by inducing BOLD near the CSF/pial surface with two different voxel resolutions (0.75 mm and 0.125 mm) (see Supplementary Methods, Simulating the large BOLD signal near CSF/pial boundary). The experimentally observed differences in BOLD signal near the CSF/pial surface between the IVE and AVF data (Fig. 3b) were reproduced by the simulations (see Supplementary Fig. S4a). Thus, the partial volume effect can be significant across depths, particularly with larger voxel sizes. With regard to b), it is often believed that masking out draining veins from the cortical depth analysis excludes signal contamination from draining veins^19,44^. However, as the magnetic field disturbances from surface blood vessels extend to the tissue voxels, this results in fMRI signal changes even in remote voxels (i.e. “blooming effect”^45,46^). For example, this results in apparent broadening of vessel diameter after injection of a contrast agent with different magnetic susceptibility. In high resolution fMRI, therefore, the laminar profiles can potentially be affected by remote pial vessels, which can influence the signal changes in 3D space.

It is important to note that the magnitude of the blooming effect depends on the spatial resolution of the voxels. Thus, the blooming effect stemming from pial veins within or outside of the imaging slice may be lower or higher in the anisotropic approach due to altered intra-voxel field inhomogeneity. Thus, whether the total blooming effect is stronger in the AVF or IVE depends on the relative location of these veins. In the current study, a lot of care was taken to place the ROI during acquisition guided by the anatomical image in order to have the largest distances to cortical surfaces outside of the imaged volume. Therefore, it is plausible to assume that with such an approach, the blooming effect due to the pial veins is reduced, compatible with our finding of smaller BOLD signal amplitudes in the upper layers of the cortex in the AVF vs. the IVE approach (see Fig. 3b). Nevertheless, a future modelling study on quantitative evaluation of the blooming effects stemming from surface veins as a function of distance^47^, orientation^20,48^ and voxel sizes in the context of depth-dependent GE-BOLD is necessary to provide further insight into this effect.

### Effective spatial resolution of the isotropic acquisition

The effective spatial resolution of the IVE acquisition was tested using simulations and explored the effect of the number of sampled cortical depths and the ROI size (along cortical distance) on the estimated cortical depth profiles by reconstructing induced PSF (FWHM~1/5 of cortical thickness in Fig. 5) with three (e.g. infragranular, granular, supragranular) and twenty one sampled cortical layers using the standard post-processing by upsampling. The results of the simulations indicate that increasing the number of voxels only minimally improves the inherent blurring of the reconstructed PSF^25^. Additionally, we can conclude that for a given spatial resolution of the functional data (assuming isotropic voxels), an ROI with number of voxels < 100 is insufficient to achieve a reasonably accurate estimate of PSF. While in practice, it is nearly impossible to reconstruct the ‘true’ width of the laminar profile with hitherto standard depth sampling approaches, a technique known as ‘spatial GLM’^26,43,49–51^ is presently under development, which accounts for the partial volume distribution between voxels spanning the cortical depths, can in theory, reconstruct the ‘true’ laminar profile almost perfectly. However, this approach requires high SNR to be reliable^43^, which can be challenging at high spatial resolutions. Alternative data analysis approaches using sub-millimetre isotropic data include sorting voxels by the relative distance of their centroids to the cortical boundaries^25,30^. This specific approach avoids interpolation into a higher spatial resolution but keeps the data in the original space, which may already be sufficient to demonstrate laminar effects of cognitive processes in some experiments. Therefore, we consider this approach to be an important, albeit under-utilised, exploration tool for high-resolution fMRI data. However, it still suffers from limitations of inferring laminar fMRI activity from an extended ROI, in contrast to single segments in the AVF approach, as voxels from many cortical segments have to be sampled in order to distinguish different depths.

### BOLD signal variability along the cortical distance

In addition, a major assumption made in the analysis of standard high-resolution fMRI data is that the layer-specific fMRI signal within the ROI only varies across cortical depth but not tangentially along the cortical distance. Thus, the AVF approach affords the unique opportunity to also investigate the BOLD variability along the cortical distance. We observed that each cortical distance segment has a strong depth-dependent BOLD response, but these are markedly different between neighbouring segments. This variability could be due to thermal noise, therefore random, or due to susceptibility-induced processes, therefore possibly meaningful. In order to investigate this, we assessed the variability of the positive BOLD signal change over trials by employing a PCA with the hypothesis that, if the tangential variability was due to physiological or susceptibility-induced processes, the first PC has to represent these profiles with high fidelity. We observed that the first PC (Fig. 4a, third panel) retains the tangential variability that we observed in individual trials (Fig. 4a, first and second panels). Although a full characterization of the nature of this variability is beyond the scope of this study, we show that the assumption of the BOLD signal homogeneity in an ROI, necessary for the isotropic data analysis, may not be accurate (see also Havlicek, *et al*.^43^) and that this tangential variability has physiological or physical origins. In other words, there may be considerable amount of tangential variability also in the isotropic acquisition, which however, may be difficult to visualize directly and take into account in the further processing of the data. At this stage, the physiological or physical correlate of the tangential variability cannot be established (except excluding random thermal noise) as, to the best of our knowledge, no electrophysiological study has been performed studying tangential variability at this spatial scale with laminar probes.

### Mapping the BOLD signal along cortical distance with AVF acquisition

Even though the main focus of the current study was the demonstration of the feasibility and superiority of the AVF acquisition for cortical depth analysis, we exploited the flexibility of the AVF acquisition strategy to also test the feasibility of sampling cortical distance with the ultra-high spatial resolution (i.e. 0.1 mm) by swapping the phase-encoding and readout directions. We identified the same patch of cortex from data with high-resolution along the cortical depth and compared the BOLD signal magnitudes to the data with high-resolution along cortical distance. We also demonstrate the tangential variability of the fMRI signal along the cortical distance and the signal averages over cortical depth and sampled signal over cortical distance corresponded very well with each other, further supporting the susceptibility origin of the tangential variability observed in the fMRI responses.

### Limitations

One limitation of using the AVF approach is the low brain coverage, which in the current study was defined by a single slice through the occipital lobe. Working with single slices can be challenging, in particular due to sensitivity to subject motion. However, in the present study, we were able to correct subject motion in all but two runs in total across all subjects and obtained significant activation in all subjects in each motion-corrected run. This was especially aided by the fact that the participants were experienced in high-resolution studies. However, this would not be the case for naïve participants and therefore, future studies employing the AVF approach may consider using prospective motion correction^52^ in addition to the manual curation during pre-processing. Please note, that additional slices can be acquired with the AVF approach and thus, extend brain coverage albeit with increasing TR (see Supplementary Fig. S2). Nevertheless, as can be seen with the resulting activation maps, acquiring more than 4 slices is currently not recommended as the tSNR falls rapidly thereafter (or data is acquired for longer durations).

Furthermore, prior knowledge of the subjects’ anatomy and the approximate locus of activation are other limitations of the AVF approach, as making informed slice positioning is critical to the accurate sampling of cortical depths. Nevertheless, this information can be obtained using a quick anatomical scan and a functional localizer. Thus, we envision that the AVF approach can be utilized in the following order: (i) Acquisition of low- or high-resolution functional localizer to determine the cortical patch of interest; (ii) Acquisition of high-resolution anatomy to guide the positioning of the anisotropic voxels; (iii) Positioning of the anisotropic slice(s) in order to zoom-in on the cortical depths or distances. We do not suggest using the anisotropic approach in an explorative way without prior knowledge of the activation locus but rather use it to study the intra-cortical processing in a known functional ROI. Insights gained from the AVF approach in this manner can help to interpret the depth-dependent or low-resolution signal changes observed with isotropic sub- or supra-millimetre acquisitions, typically used in cognitive neuroscience applications. Given that the activation locus is known before the placement of the AVF slice(s), the orientation of the slice(s) can be optimized in a way such that the maximum number of depths or tangential distances is probed.

Lastly, the application of the AVF acquisition approach as presented in the current study is limited to regions with relatively flat patches of cortex due to the anisotropicity. However, even a convoluted cortex is locally flat (i.e. its curvature can be locally approximated by a first order Taylor expansion). Therefore, to be able to apply the AVF method using the same dimensions as in the paper, a relatively flat cortex for about ~1.4 mm × 2 mm is required, which is reasonably fulfilled in any part of the human brain. Additionally, applying AVF in areas with curvature just effectively reduces the number of cortical depths sampled, which however, may still be sufficient for imaging laminar processing (e.g. instead of ~30 cortical depths in flat part of the cortex, ~20 depths may still be discerned with oblique orientations).

The AVF approach is presently only applicable to T_2_*-dependent studies. Given that GE-BOLD remains the workhorse for most laminar fMRI studies, we believe that the AVF approach can be a useful add-on method to aid interpretability in such studies. In combination with modelling of the non-local BOLD signal due to ascending veins^32^, we expect that the AVF approach presented in this study will allow studying functional laminar organization of the human cortex at an unprecedented spatial detail, currently only possible with invasive methods, such as laminar probes used in electrophysiology.

## Methods

Seven healthy volunteers (median age = 28 years, 3 female) participated in the study following screening and having given written informed consent. The study was approved by the Ethics Review Committee for Psychology and Neuroscience (ERCPN) at Maastricht University and all procedures followed the principles expressed in the Declaration of Helsinki. All data were acquired on a whole-body Magnetom 7T research scanner (Siemens Healthineers, Erlangen, Germany) using a custom-built 16 Rx channel phased-array visual cortex coil (see Supplementary Fig. S1)^37^.

Before scanning, the ROI was identified using the subject’s anatomical scan from a previous MRI session (see Supplementary Methods, Region-of-interest). In a scan session, a high-resolution (0.7 mm isotropic) MP2RAGE localizer was acquired to position the acquisition slab accurately over the ROI and oriented such that the phase-encoding direction was orthogonal to the 0.1 mm spatial resolution direction. Three functional runs were acquired with the anisotropic voxel FLASH (AVF) (0.1 × 1.4 × 2.0 mm^3^). The same acquisition orientation was used to acquire two functional runs with the isotropic voxel EPI (IVE) (0.7 mm isotropic). To facilitate laminar analysis in native EPI space, we acquired a MI-EPI^21^ (0.7 mm isotropic) as a distortion-matched anatomical reference to the IVE scans. Additional functional data was acquired in one subject in a separate session using the AVF approach, sampling cortical depth (laminar direction) and cortical distance (columnar direction) with the 0.1 mm resolution in alternate runs. In the same session, functional AVF data was also acquired with increasing coverage (2, 4 and 6 slices). Sequence-specific details and acquisition parameters are fully described in Supplementary Methods and listed in see Supplementary Table S1, respectively.

All data analysis was performed at the single subject level independently for the AVF and IVE datasets (see Supplementary Methods). The laminar time-courses were extracted and then averaged for comparison. Variability along the cortical distance was assessed using a PCA (see Supplementary Methods, Variability along cortical distance) over trials using MATLAB in the native data resolution. The effective spatial resolution of conventional IVE acquisitions (see Supplementary Methods, Effective spatial resolution) and the partial volume effect of BOLD activation at the CSF/pial boundary with two different voxel sizes (see Supplementary Methods, Simulating the large BOLD signal near the CSF/pial boundary) were evaluated by simulating an anatomically-inspired cortex with variable cortical thickness (1.7–3.7 mm)^43^.

## Electronic supplementary material

Supplementary Information

## Data Availability

The data from the current study are available from the corresponding authors on request.
