# Supplementary material for: Resolving laminar activation in human V1 using ultra-high spatial resolution fMRI at 7T
Source: Sci Rep. 2018 Nov 20;8:17063. doi: 10.1038/s41598-018-35333-3 (PMC6244001; doi:10.1038/s41598-018-35333-3)
Supplement: Supplementary file 1 — Supplementary Information [file 41598_2018_35333_MOESM1_ESM.pdf]

Supplementary Information

Resolving laminar activation in human V1 using ultra-high spatial resolution fMRI at 7T

Sriranga Kashyap*[1], Dimo Ivanov[1], Martin Havlicek[1], Shubharthi Sengupta[1], Benedikt A. Poser[1], Kâmil Uludağ*[1,2]

[1] Department of Cognitive Neuroscience, Faculty of Psychology and Neuroscience, Maastricht University, 6229 EV Maastricht, Netherlands

[2] Center for Neuroscience Imaging Research, Institute for Basic Science (IBS), and Department of Biomedical Engineering, Sungkyunkwan University, Suwon 16419, Republic of Korea

Supplementary Methods

Participants

Seven healthy volunteers (median age=28 years, 3 female) participated in the study following screening and having given written informed consent. The study was approved by the Ethics Review Committee for Psychology and Neuroscience (ERCPN) at Maastricht University and all procedures followed the principles expressed in the Declaration of Helsinki.

Stimulus paradigm

Full contrast black-and-white radial flickering (~8 Hz) checkerboard was presented using PsychoPy [1,2] for 20 s (stimulus on) followed by 40 s of an isoluminant grey background (stimulus off). Each functional run lasted ~12 minutes consisting of a 30 s initial baseline period and ten stimulus on-off blocks. The participants were instructed to remain motionless and fixate on a central fixation dot throughout each functional run.

Region-of-interest (ROI)

Similar to the line-scanning approach [3], the cortical patch selected as ROI was determined from a continuous 'flat' portion of the subject's calcarine sulcus (therefore, V1), with additional criterion that the ROI was approximately uniformly thick and stayed flat for about 2.0 mm along the slice direction. The ROIs were first identified in each subject based on an anatomical MP2RAGE reference scan from a previous MRI session to save time during the acquisition session of the fMRI data. Based on this prior knowledge of the ROI location, the session-specific MP2RAGE localizer was used to fine-tune the placement of the fMRI imaging volume.

Data acquisition

All data were acquired on a whole-body Magnetom 7T research scanner (Siemens Healthineers, Erlangen, Germany) using a custom-built 16 Rx channel phased-array visual cortex coil

(Supplementary Fig. S1) [4]. A single session consisted of a total of three anatomical scans (MP2RAGE localizer, MP2RAGE reference, MI-EPI reference) and a total of five functional scans. The sequence parameters, listed in Supplementary Table 1, were optimized in pilot experiments.

In each session, a high-resolution MP2RAGE [5] (0.7 mm isotropic) was acquired first and used as an anatomical localizer for slice planning. The strong tissue contrast of the MP2RAGE was critical for positioning the imaging volume on the ROI for the functional scans. Three functional runs were acquired using an inhouse adapted FLASH [6] with anisotropic spatial resolutions (0.1x1.4x2.0 mm^3) (called Anisotropic Voxel FLASH, abbreviated as AVF) along the readout and phase-encoding (matrix size 1200x86) directions. Factor of two GRAPPA undersampling was used, resulting in a total of 43 k-space lines being acquired per slice at TR=41 ms (total slice TR=1.76 s) and a flip angle of 12°. No partial Fourier was used to minimize blurring. At the echo time (TE) of 26 ms, a readout bandwidth of 60 Hz per pixel could be afforded in order to maximize SNR. In addition, RF excitation with high bandwidth-time product (8.5) was applied to reduce slice profile imperfections. The high spatial resolution (0.1 mm) along the readout direction was selected so as to sample cortical depths, and therefore positioned orthogonal to the cortical surface in the ROI. The precise slice placement and orientation was thus adapted on a subject-by-subject basis, based on the curvature of the calcarine sulcus, such that the phase-encoding direction is locally along the cortical distance (see Figure 1). This is critical to prevent any phase-encoding dependent blurring from occurring across the cortical depths in the ROI. A second MP2RAGE with a high in-plane spatial resolution (0.4x0.4x0.7 mm^3) was acquired with the same orientation as the AVF acquisitions. Subsequently, two functional runs were obtained using gradient-echo 3D-EPI [7] with isotropic spatial resolution (0.7mm isotropic) (called Isotropic Voxel EPI, abbreviated as IVE), with the same FoV orientation as the AVF fMRI scans. Lastly, a multiple inversion recovery time EPI (MI-EPI) (0.7mm isotropic) with 64 inversion times [8,9] was acquired for anatomical reference, with the distortions matched to the isotropic 3D-EPI functional scans.

In order to further study the properties of the AVF acquisition, one of the seven subjects participated in an additional fMRI session using the same stimulus paradigm as in the main study. In this session, a high-resolution MP2RAGE was acquired as an anatomical localizer and was followed by an AVF run with highest-resolution sampling the cortical depth (Figure 6a, left). The phase-encoding and frequency-encoding directions were then switched for the second and third functional runs, achieving the highest spatial resolution (0.1mm) along the cortical distance, thereby sampling cortical “columns” (Figure 6a, right). Three additional functional runs were obtained using the AVF with laminar resolution

but with increasing number of slices (i.e. increasing coverage), which resulted in consequently longer TRs [Run 1: Slices = 2, TR_{eff} = 3.53 s; Run 2: Slices = 4, TR_{eff} = 7.05 s; Run 3: Slices = 6, TR_{eff} = 10.58 s] (Figure S2). All other sequence parameters remained the same as in the main study (Table S1).

Data processing and analysis

Functional data

All AVF runs were motion-corrected with Advanced Normalization Tools (ANTs, https://github.com/ANTsX/ANTs) [10,11] using the time-series mean as the reference volume (*Translation, antsRegistration*). Each motion-corrected functional run was then carefully examined for realignment artefacts, and anatomical features, such as veins (low intensity voxels), were tracked on a volume-by-volume basis as a quality check. Statistical analysis of the fMRI data was done using FEAT 6.0 (FMRI Expert Analysis Tool), part of FSL (FMRIB's Software Library, https://fsl.fmrib.ox.ac.uk/fsl/fslwiki) [12]. The grand-mean normalized time-series was temporally filtered ($\sigma_{high\text{-}pass}$=25xTR, $\sigma_{low\text{-}pass}$=1.5xTR) and statistical analysis was carried out using FILM with local autocorrelation correction [13]. The GLM model included a double gamma HRF with temporal derivatives and motion parameters as nuisance regressors. The within-subject higher-level analysis was carried out using a fixed effects model by forcing the random effects variance to zero in FLAME (FMRIB's Local Analysis of Mixed Effects) [14,15]. The quantitative T_1 map from the second MP2RAGE (acquired with the same orientation as the AVF) was registered to the motion-corrected time-series mean AVF fMRI image using the registration tools in ITK-SNAP 3.6 (http://www.itksnap.org) [16]. Note that, in contrast to standard analysis in low-resolution fMRI studies, the anatomy was registered to the functional data in order to keep the functional data in its original space and, hence, avoid any additional smoothing [9]. The images were first aligned using the mutual information cost-function, and further manual fine-tuning of the alignment was performed using ITK-SNAP's interactive registration tool. The transformation matrix was exported in the ITK format and was applied to the anatomical data using ANTs (*antsApplyTransforms, 4th order B-spline interpolation*).

All IVE runs were also motion-corrected with ANTs using the time-series mean as the reference volume (*Rigid, antsRegistration*). No spatial smoothing or distortion-correction was applied (see MI-EPI workflow in Kashyap, et al. [9]). Further statistical analysis of the fMRI data was done using FEAT 6.0, as described above. A distortion-matched T_1 map was computed by fitting the anatomical MI-EPI data [9].

Segmentation of anatomical images

ITK-SNAP 3.6 was used to semi-automatically segment the subject-specific MP2RAGE and MI-EPI T_1 maps into three tissue classes based on T_1 signal intensity: grey-matter (GM), white-matter (WM) and cerebrospinal fluid (CSF). These binary tissue class masks were manually corrected in ITK-SNAP after co-registration to the functional data, to ensure accurate delineation of the boundaries in the ROI and fix any co-registration artefacts.

Cortical depth analysis

The AVF fMRI time-series data was imported into MATLAB R2015b (Mathworks, USA) for laminar analysis. The average GM thickness in the V1 ROIs across our subjects was ~3 mm, i.e. 30 voxels with 0.1 mm extent in the cortical depth direction. In order to be able to average cortical depth profiles over subjects with varying GM thickness, we linearly interpolated the total number of sampled cortical depths to 30 in each subject's ROI. For the IVE dataset, cortical depth analysis was done using CBS-Tools [17]. The CSF-GM and WM-GM boundary masks from the MI-EPI T_1 segmentation were first converted to the levelset representation, and 30 equidistant [18,19] depths were defined in GM. The equidistant model of cortical layering was selected as it approximates to the equi-volumnar model because our ROIs did not include regions of high cortical curvature [20].

To check whether similar results are obtained in both acquisition types, if the same ROI is considered, the cortical depth timecourses were averaged along the direction of the cortical distance for all subjects for both AVF and IVE acquisitions. The mean of the positive BOLD and post-stimulus undershoot signals were calculated by averaging over the time windows 12-26 s and 32-42 s following stimulus onset, respectively. The event-related cortical depth timecourses from both datasets were normalized to the mean positive BOLD signal change between 30-70% of cortical depth of each ROI for all subjects, in order to minimize bias to subjects with high BOLD signal changes. The normalized event-related timecourses were then averaged across subjects (Figure 3a). The cortical depth analysis was always carried out using the data across the entire ROI (i.e. no statistical threshold or masking was used).

Variability along cortical distance

Typically, in laminar fMRI studies [21-25], the sampled depth-specific signals are averaged tangentially along the cortical distance in an ROI. The principle reason for this being, due to the relatively large voxels (~0.7-0.8mm) relative to the histological layer thicknesses, the underlying laminar signals have to be inferred by averaging upsampled fMRI data and accounting for each voxel's partial volumes across the different depths (*see section: Effective spatial resolution, below*). Due to this limitation, most studies are unable to assess the tangential variability of the acquired signal. In the AVF fMRI data, such upsampling and averaging over an ROI is in principle not necessary, assuming sufficient tSNR. Thus, we assessed the reliability of the AVF fMRI acquisition to probe the tangential variability using Principal Component Analysis (PCA) on the positive BOLD profiles in our ROI with the hypothesis that, if the profiles are similar over trials, then a PCA decomposition over trials should result in most of the variance in the data being explained by the First principal component (PC). For comparison, the PCA was also carried out for the IVE data in its native resolution.

Effective Spatial-Resolution

It is generally assumed that, using the IVE fMRI data, upsampling and averaging over an extensive ROI can in principle yield super-resolved cortical depth profiles with effective spatial resolution only being limited by the extent of the ROI. In order to test this assumption and compare the effective spatial resolution of the IVE with the AVF acquisition, we simulated an anatomically-inspired sample of visual cortex with variable cortical thicknesses (1.7-3.7 mm) at 0.125 mm isotropic resolution [26] (Supplementary Fig. S3a). Twenty-one equi-volumnar cortical depths were defined, and voxel-partial-volumes were calculated for each cortical depth. Equi-volumnar model of layering was chosen here because of regions of high-curvature in the simulated cortex (Supplementary Fig. S3b). Two fMRI datasets were generated assuming a Gaussian shaped activation profile in the middle of cortical ribbon with a full-width-at-half-maximum (FWHM) ~1/5th (Figure 5) of the cortical thickness. The data was then downsampled (by averaging) to a 0.75 mm isotropic resolution. Next, we attempted to reconstruct the original activity profile by sampling the signal with three and twenty-one calculated cortical depths, respectively. Prior to layer sampling, the simulated functional data was upsampled using a nearest-neighbour interpolation method to 0.125 mm resolution and partial volume maps were calculated at this resolution for three and twenty-one numbers of cortical depths, respectively.

Signal intensities of small voxels with corresponding partial volume greater than 0.75 within the cortical depth were averaged.

We evaluated (a) the effect of the number of voxels (5-200) within an ROI with randomly assigned location along the cortical ribbon; and (b) the effect of additive noise, with SNR (σ_S/σ_N) = 1 (i.e. realistic) and infinite (i.e. ideal) for two sampled number of cortical depth and widths of the activation profile. The point spread functions (PSF) were first reconstructed and the overlapping area under the true PSF was calculated to quantify blurring induced by subsampling the original signal. All simulations were repeated 200 times for both activation profile widths.

Simulating the large BOLD signal near the CSF/pial boundary

To simulate the effect of leakage of the signal change in the pial vein to the cortical layers due to remaining partial volume effects, we considered activation (amplitude = 5) in the CSF/pial boundary and the very first cortical depth (1st of 21). The remaining lower 20 cortical depths were considered not to be activated. Two scenarios, with larger (0.75 mm isotropic) and smaller (0.125 mm isotropic) voxel sizes, were compared. This represents a simplified analogy for the acquisition strategies (IVE and AVF) and their differences in laminar spatial resolution. The induced laminar profile (Supplementary Figure S4) was reconstructed by sampling the signal from twenty-one cortical depths and excluding regions with high-curvature (normalised curvature>0.3), having performed 100 repetitions of Monte Carlo simulation for noise-less (SNR_{ideal} = infinite, Supplementary Figure S4a) and additive noise ($SNR_{realistic}$ = 1, Supplementary Figure S4b) cases. Additionally, simulations were repeated using only regions of high-curvature (normalised curvature>0.3). For larger voxels (0.75 mm), the estimated signal amplitude is lower at the surface and drops slowly, i.e. extending to 40% of cortical depth. For smaller voxels (0.125 mm), the estimated signal amplitude recovers the true amplitude level at the surface boundary and drops quickly within less than 20% of cortical depth. The reconstructed profiles (between 10-100% of cortical depth) in Supplementary Figure S4b are very comparable to the experimental results in Figure 3 for the two acquisition approaches.

If a convolution kernel, derived for 0.75 mm isotropic voxels as per Koopmans et al.[27], is applied to the induced laminar activation profile, the resulting reconstructed profile is very similar to the reconstructed profile obtained from the Monte Carlo simulations with 0.75 mm voxels (see Supplementary Figure S4a, red dashed line). In the case for high-curvature regions, the laminar profile

for 0.75 mm voxels extends to deeper cortical depths (~50%), while the laminar profile reconstructed using 0.125 mm voxels is nearly identical to that obtained from the regions excluding high-curvature regions (Supplementary Figure S4b).

Supplementary References

1 Peirce, J. W. Generating stimuli for neuroscience using PsychoPy. *Frontiers in neuroinformatics* **2**, 10-10 (2009).

2 Peirce, J. W. PsychoPy-Psychophysics software in Python. *Journal of Neuroscience Methods* **162**, 8-13, doi:10.1016/j.jneumeth.2006.11.017 (2007).

3 Yu, X., Qian, C., Chen, D. Y., Dodd, S. J. & Koretsky, A. P. Deciphering laminar-specific neural inputs with line-scanning fMRI. *Nat Methods* **11**, 55-58, doi:10.1038/nmeth.2730 (2014).

4 Sengupta, S. *et al.* A Specialized Multi-Transmit Head Coil for High Resolution fMRI of the Human Visual Cortex at 7T. *PLOS ONE* **11**, e0165418, doi:10.1371/journal.pone.0165418 (2016).

5 Marques, J. P. *et al.* MP2RAGE, a self bias-field corrected sequence for improved segmentation and T1-mapping at high field. *NeuroImage* **49**, 1271-1281, doi:10.1016/j.neuroimage.2009.10.002 (2010).

6 Haase, A., Frahm, J., Matthaei, D., Hanicke, W. & Merboldt, K. D. FLASH imaging. Rapid NMR imaging using low flip-angle pulses. *Journal of Magnetic Resonance (1969)* **67**, 258-266, doi:10.1016/0022-2364(86)90433-6 (1986).

7 Poser, B. A., Koopmans, P. J., Witzel, T., Wald, L. L. & Barth, M. Three dimensional echo-planar imaging at 7 tesla. *NeuroImage* **51**, 261-266, doi:10.1016/j.neuroimage.2010.01.108 (2010).

8 Renvall, V., Witzel, T., Wald, L. L. & Polimeni, J. R. Automatic cortical surface reconstruction of high-resolution T1echo planar imaging data. *NeuroImage* **134**, 338-354, doi:10.1016/j.neuroimage.2016.04.004 (2016).

9 Kashyap, S., Ivanov, D., Havlicek, M., Poser, B. A. & Uludağ, K. Impact of acquisition and analysis strategies on cortical depth-dependent fMRI. *NeuroImage*, doi:10.1016/j.neuroimage.2017.05.022 (2018).

10 Avants, B. B. *et al.* A reproducible evaluation of ANTs similarity metric performance in brain image registration. *Neuroimage* **54**, 2033-2044, doi:10.1016/j.neuroimage.2010.09.025 (2011).

11 Avants, B. B., Tustison, N. J., Wu, J., Cook, P. A. & Gee, J. C. An open source multivariate framework for n-tissue segmentation with evaluation on public data. *Neuroinformatics* **9**, 381-400, doi:10.1007/s12021-011-9109-y (2011).

12 Jenkinson, M., Beckmann, C. F., Behrens, T. E. J., Woolrich, M. W. & Smith, S. M. Fsl. *NeuroImage* **62**, 782-790, doi:10.1016/j.neuroimage.2011.09.015 (2012).

13 Woolrich, M. W., Ripley, B. D., Brady, M. & Smith, S. M. Temporal autocorrelation in univariate linear modeling of FMRI data. *Neuroimage* **14**, 1370-1386, doi:10.1006/nimg.2001.0931 (2001).

14 Woolrich, M. W., Behrens, T. E., Beckmann, C. F., Jenkinson, M. & Smith, S. M. Multilevel linear modelling for FMRI group analysis using Bayesian inference. *Neuroimage* **21**, 1732-1747, doi:10.1016/j.neuroimage.2003.12.023 (2004).

15 Beckmann, C. F., Jenkinson, M. & Smith, S. M. General multilevel linear modeling for group analysis in FMRI. *Neuroimage* **20**, 1052-1063, doi:10.1016/S1053-8119(03)00435-X (2003).

16 Yushkevich, P. A. *et al.* User-guided 3D active contour segmentation of anatomical structures: significantly improved efficiency and reliability. *Neuroimage* **31**, 1116-1128, doi:10.1016/j.neuroimage.2006.01.015 (2006).

17 Bazin, P. L. *et al.* A computational framework for ultra-high resolution cortical segmentation at 7Tesla. *Neuroimage* **93 Pt 2**, 201-209, doi:10.1016/j.neuroimage.2013.03.077 (2014).

18 Leprince, Y., Hasboun, D., Poupon, F., Poupon, C. & Rivi, D. Combined Laplacian-Equivolumic Model For Studying Cortical Lamination With Ultra High Field MRI (7T). 580-583, doi:10.1109/ISBI.2015.7163940 (2015).

19 Waehnert, M. D. *et al.* Anatomically motivated modeling of cortical laminae. *Neuroimage* **93 Pt 2**, 210-220, doi:10.1016/j.neuroimage.2013.03.078 (2014).

20 Kemper, V. G., De Martino, F., Emmerling, T. C. T. C., Yacoub, E. & Goebel, R. High resolution data analysis strategies for mesoscale human functional MRI at 7 and 9.4 T. *NeuroImage* **164**, 48-58, doi:10.1016/j.neuroimage.2017.03.058 (2018).

21 Fracasso, A., Luijten, P. R., Dumoulin, S. O. & Petridou, N. Laminar imaging of positive and negative BOLD in human visual cortex at 7T. *Neuroimage* **164**, 100-111, doi:10.1016/j.neuroimage.2017.02.038 (2018).

22 Huber, L. *et al.* High-resolution CBV-fMRI allows mapping of laminar activity and connectivity of cortical input and output in human M1. *Neuron* **96**, 1-11, doi:10.1016/j.neuron.2017.11.005 (2017).

23 Marquardt, I., Schneider, M., Gulban, O. F., Ivanov, D. & Uludag, K. Cortical depth profiles of luminance contrast responses in human V1 and V2 using 7 T fMRI. *Hum Brain Mapp*, doi:10.1002/hbm.24042 (2018).

24 Polimeni, J. R., Fischl, B., Greve, D. N. & Wald, L. L. Laminar analysis of 7T BOLD using an imposed spatial activation pattern in human V1. *NeuroImage* **52**, 1334-1346, doi:10.1016/j.neuroimage.2010.05.005 (2010).

25 Koopmans, P. J., Barth, M. & Norris, D. G. Layer-specific BOLD activation in human V1. *Human Brain Mapping* **31**, 1297-1304, doi:10.1002/hbm.20936 (2010).

26 Havlicek, M., Kashyap, S., Ivanov, D. & Uludag, K. Towards an optimal analysis of laminar-resolved fMRI. *Proceedings of OHBM, Geneva, Switzerland* (2016).

27 Koopmans, P. J., Barth, M., Orzada, S. & Norris, D. G. Multi-echo fMRI of the cortical laminae in humans at 7T. *NeuroImage* **56**, 1276-1285, doi:10.1016/j.neuroimage.2011.02.042 (2011).

28 Polimeni, J. R. *et al.* Reducing sensitivity losses due to respiration and motion in accelerated echo planar imaging by reordering the autocalibration data acquisition. *Magnetic resonance in medicine* **75**, 665-679, doi:10.1002/mrm.25628 (2016).

Supplementary Figures and Table

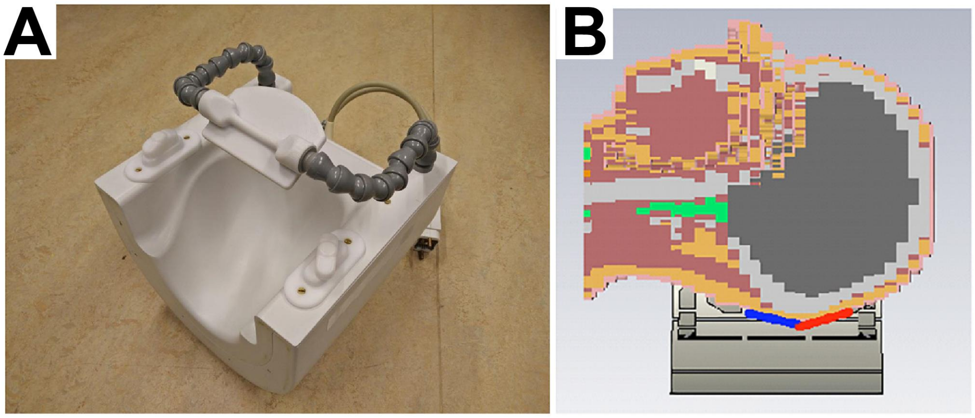

Supplementary Figure S1: Specialized visual cortex RF coil array used in this study. (A) Assembled coil with mounted wide field-of-view mirror; (B) Two rows each of eight receive coil elements, (indicated in blue and red) encapsulate the occipital lobe (Adapted from Sengupta, et al. [4] with permission).

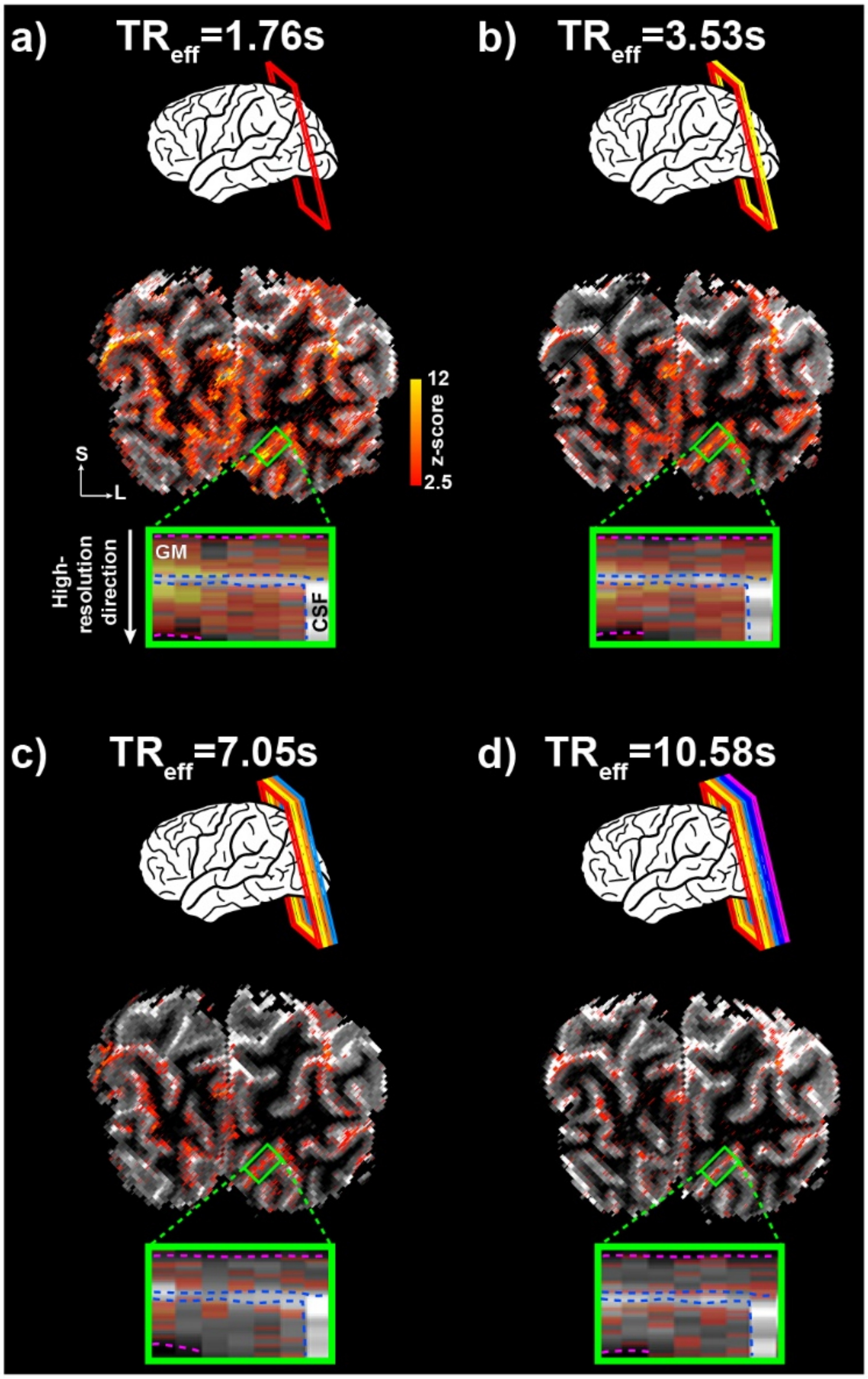

Supplementary Figure S2: Single-subject single-run activation maps overlaid on an MP2RAGE T_1 map demonstrating the different acquisition schemes with multiple slices having highest spatial resolution along the laminar direction using the anisotropic 2D-FLASH approach.

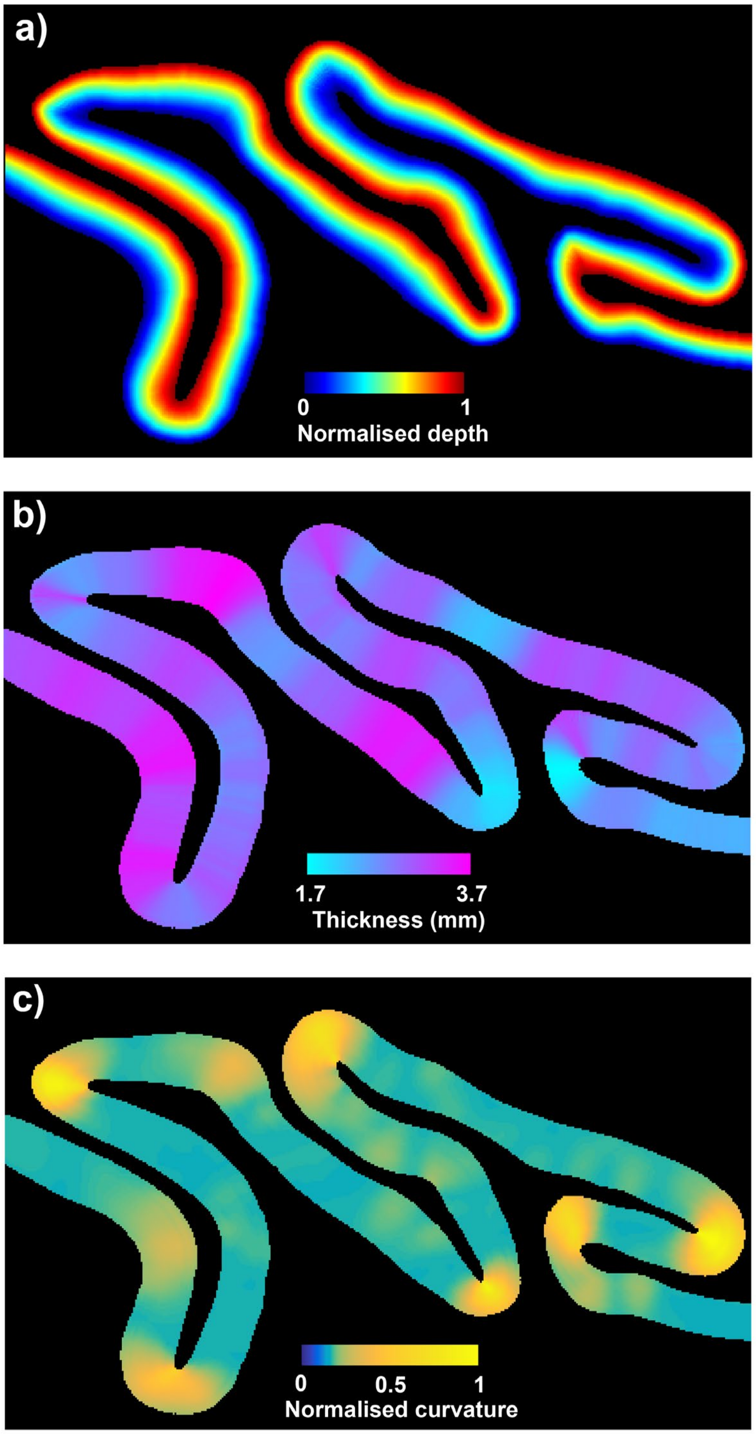

Supplementary Figure S3: (a) Equi-volumnar layering, (b) variable cortical thickness and (c) normalised curvature in the anatomically-inspired patch of cortex used for simulations.

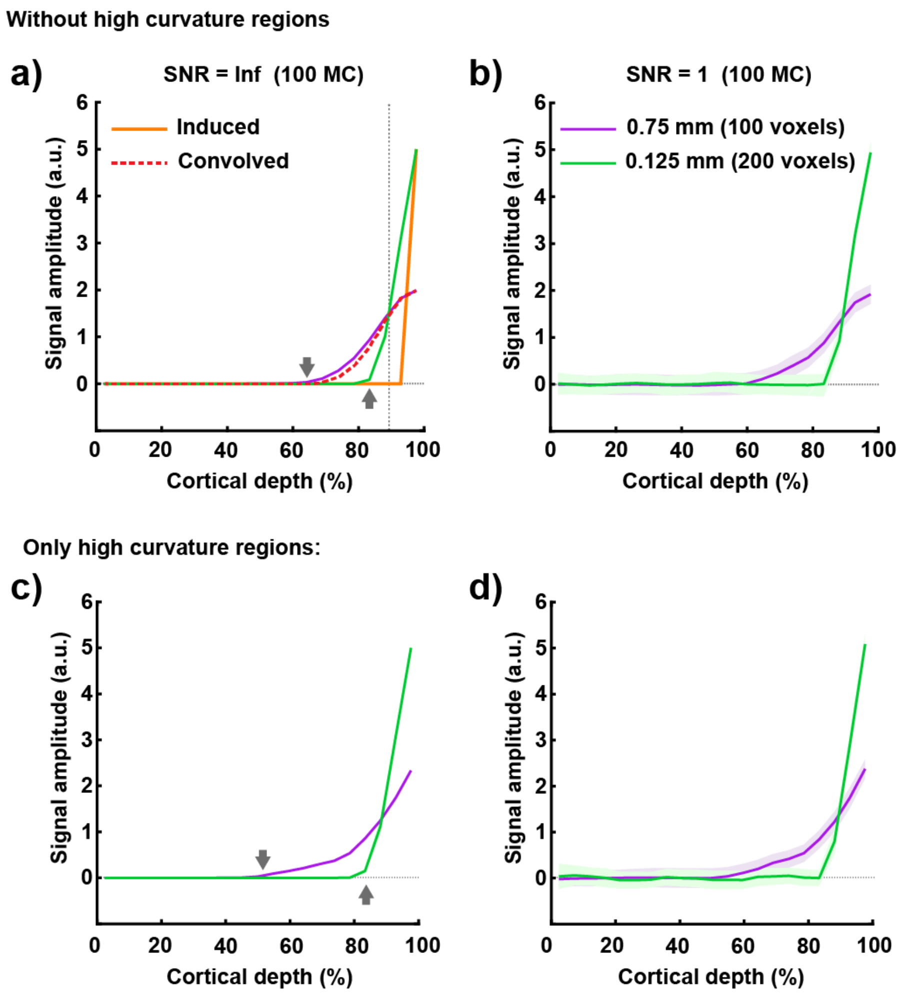

Supplementary Figure S4: (a) The induced activation (amplitude=5) in the uppermost cortical depth, plotted together with the reconstructed laminar profiles sampled from patches of cortex excluding high cortical curvature, obtained with 0.75mm (purple) and 0.125mm (green) isotropic voxels, respectively for the case of infinite SNR following 100 Monte-Carlo simulations. The red dotted line represents the induced laminar profile convolved with a convolution kernel derived for the 0.75mm voxels as described in Koopmans et al.[27]. (b) Reconstructed laminar profiles for the different voxel sizes for the case of realistic SNR following 100 Monte Carlo simulations. (c-d) Reconstructed laminar profiles sampled from regions of high cortical curvature for the different voxel sizes for the case of infinite and realistic SNR, respectively following 100 Monte-Carlo simulations.

Supplementary Table S1. Imaging parameters used for data acquisition.

Sequence	Resolution (mm^3)	Matrix size	Other parameters
**3D-MP2RAGE [5] (localizer)**	0.7x0.7x0.7	320x320	FoV=224x224 mm^2; 240 sagittal slices; GRAPPA=3; partial Fourier=6/8; Ref. lines PE=24; TR=5000 ms; TE=2.47 ms; TI_1/TI_2=900/2750 ms; α_1/α_2=5°/3°; Bandwidth (BW)=250 Hz/pixel; echo-spacing=6.9 ms
**2D-FLASH [6] (fMRI - AVF)**	0.1x1.4x2.0	1200x86	FoV=120x120 mm^2; GRAPPA=2; Ref. PE lines=64; TR_{eff}=1763 ms; TE=26 ms; α=12°; BW=60 Hz/pixel; RF bandwidth-time product (BWTP)=8.5; RF Duration=3000 μs
**3D-MP2RAGE (anatomy)**	0.4x0.4x0.7	320x320	FoV=120x120 mm^2; 240 sagittal slices; GRAPPA=3; partial Fourier=6/8; Ref. lines PE=24; TR=5000 ms; TE=2.42ms; TI1/TI2=900/2750 ms; α1/α2=5°/3°; BW=280 Hz/pixel; echo-spacing=7 ms
**3D-EPI [7] (fMRI - IVE)**	0.7x0.7x0.7	214x214	FoV=150x150 mm^2; GRAPPA=3; TR_{eff}=1890 ms; TE=26 ms; Ref. lines PE=78; Ref. lines 3D=32; phase partial Fourier=6/8; slice partial Fourier=6/8; α=16°; BW=1016 Hz/pixel; echo-spacing=1.09 ms; PAT refscan mode=FLASH
**MI-EPI [8,9] (anatomy)**	0.7x0.7x0.7	214x214	FoV=150x150 mm^2; GRAPPA=3; TR=9000 ms; TE=29 ms; Ref. PE lines=63; phase partial Fourier=6/8; α=90°; BW=1016 Hz/pixel; echo-spacing=1.09 ms; TI_1/TI_2=50/175 ms; TIs=64; Slab-size=2; scan-time-per-slice=81.75 ms; Fat saturation; PAT refscan mode=FLEET[28]; α_{FLEET}=10°
